# Calculated parenteral initial treatment of bacterial infections: Infections with multi-resistant Gram-negative rods – ESBL producers, carbapenemase-producing Enterobacteriaceae, carbapenem-resistant Acinetobacter baumannii 

**DOI:** 10.3205/id000048

**Published:** 2020-03-26

**Authors:** Béatrice Grabein, Michael Ebenhoch, Ernst Kühnen, Florian Thalhammer

**Affiliations:** 1 Stabsstelle Klinische Mikrobiologie und Krankenhaushygiene, Klinikum der Universität München, Munich, Germany; 2 Stabsstelle Hygiene, Klinische Infektiologie und Mikrobiologie, BG-Unfallklinik Murnau, Germany; 3 Mikrobiologie & Hygiene, MVZ Synlab Trier, Germany; 4 Klinische Abteilung für Infektiologie und Tropenmedizin, Medizinische Universität Wien, Vienna, Austria

## Abstract

This is the sixteenth chapter of the guideline “Calculated initial parenteral treatment of bacterial infections in adults – update 2018” in the 2^nd^ updated version. The German guideline by the Paul-Ehrlich-Gesellschaft für Chemotherapie e.V. (PEG) has been translated to address an international audience.

Infections due to multiresistant Gram-negative rods are challenging. In this chapter recommendations for targeted therapy for infections caused by ESBL-producing Enterobacteriaceae, carbapenemase-producing Enterobacteriaceae and carbapenem-resistant *Acinetobacter baumannii* are given, based on the limited available evidence.

## Zusammenfassung

Dies ist das sechzehnte Kapitel der von der Paul-Ehrlich-Gesellschaft für Chemotherapie e.V. (PEG) herausgegebenen S2k Leitlinie „Kalkulierte parenterale Initialtherapie bakterieller Erkrankungen bei Erwachsenen – Update 2018“ in der 2. aktualisierten Fassung.

Infektionen durch resistente gramnegative Erreger sind eine Herausforderung. In diesem Kapitel werden Therapieempfehlungen für die gezielte Therapie von Infektionen durch ESBL-bildende Enterobacteriaceae, Carbapenemase-bildende Enterobactaeriaceae und Carbapenem-resistente *Acinetobacter baumannii* gegeben, die auf der begrenzten verfügbaren Evidenz beruhen.

## Antibiotics for the treatment of infections with MRGN

### Amoxicillin/clavulanic acid and piperacillin/tazobactam

By definition, clavulanic acid inhibits ESBL-positive enterobacteria in vitro and has higher beta-lactamase inhibitor (BLI) activity compared to sulbactam [1]. Tazobactam has a stronger inhibitory activity than clavulanic acid and sulbactam against ESBL but almost always exhibits inadequate inhibitory activity against carbapenemases [2]. Recent studies show that amoxicillin/clavulanic acid or piperacillin/tazobactam can be used to treat infections with ESBL-producing Enterobacteriaceae which have shown sensitivity in vitro [3], [4]. However, an inoculum effect must be taken into account, which is more pronounced in piperacillin/tazobactam than in amoxicillin/clavulanic acid [5], [6], [7]. As a logical consequence, separate limits for amoxycillin/clavulanic acid for urinary isolates of *Escherichia* *coli* have been introduced in the current EUCAST guidelines [8]. It should also be noted that different ESBL types exist in different countries and regions. The clinical data mainly refer to types that are widespread in Spain and Italy. The extent to which these data can be transferred to current conditions in Germany or Austria is not clear.

### Temocillin

Temocillin, introduced in 1988, is a semisynthetic 6-α-methoxy derivative of ticarcillin which is active against many enterobacteria but not against non-fermenters, Gram-positive aerobes and anaerobes. The methoxy group causes temocillin to target numerous beta-lactamases [9], including ESBL [10], [11], AmpC [12] and *Klebsiella* *pneumoniae* carbapenemases (KPC) but is not stable against metallo-beta-lactamases and OXA-48. The standard dosage is 2x 2 g and the maximum permissible dosage 3x 2 g temocillin. A recently published study recommends a daily dose of 6 g of temocillin for critically ill patients either intermittently three times a day or continuously after a loading dose of 2 g of temocillin [13]. An English study with a broad range of indications (urinary tract infection, bacteraemia, pneumonia) confirms the efficacy of temocillin in ESBL- and AmpC-positive enterobacterial infections [14]. Temocillin is approved in Belgium, France and the UK but not in Germany, Austria and Switzerland. In justified cases, individual import is possible according to §73 Sect. 3 Medicinal Products Act.

### Avibactam

Avibactam is the first member of a new class of non-beta-lactam beta-lactamase inhibitors. These BLIs are more potent and broader acting than tazobactam. They also include Ambler class A beta-lactamases (including carbapenemases such as KPC), C (AmpC), and D (Table 1 (Tab. 1)) [15], [16]. Avibactam is already approved in combination with ceftazidime and, used in combination with ceftaroline or aztreonam, is currently being checked in studies.

### Ceftazidime/avibactam

The fixed combination ceftazidime/avibactam also has an approximately four times stronger effect on *Pseudomonas* *aeruginosa* than ceftazidime alone due to the above-mentioned beta-lactamase activity against AmpC-beta-lactamases [17]. Due to the replacement of an amino acid in the carbapenemase KPC-2, resistance to ceftazidime/avibactam has already been observed in *Escherichia coli* [3]. The recommended dosage is 3x 2.5 g ceftazidime/avibactam corresponding to a ratio of 2 g ceftazidime to 0.5 g avibactam. There are also phase 1 study data on 3x 4 g ceftazidime/avibactam (3 g + 1 g) [18]. The elimination of both drugs is exclusively renal, which is why appropriate dose adjustments in cases of renal impairment are necessary. The concentration of both substances in the epithelial lining fluid (ELF) was about 30% of the plasma concentration. Approval has been granted for complicated intra-abdominal infections, complicated urinary tract infections including pyelonephritis, nosocomial pneumonia, including ventilator-associated pneumonia and treatment of infections with aerobic Gram-negative pathogens in adult patients with limited treatment options. 

### Ceftolozane/tazobactam

Ceftolozane is structurally similar to ceftazidime but differs from ceftazidime in the side chain at position 3 [19]. This results in a strong binding to the penicillin-binding proteins, which are responsible for the high activity against *Pseudomonas aeruginosa*, including multiply resistant strains [20]. The fixed combination is also effective against ESBL-positive enterobacteria but not against AmpC and carbapenemase-producing strains (Table 2 (Tab. 2)) [21], [22]. Ceftolozane/tazobactam is approved for the treatment of intra-abdominal infections (in combination with metronidazole) [23] as well as complicated urinary tract infections and pyelonephritis [24]. The approved dose of ceftolozane/tazobactam (in a ratio of 2:1) is 3x 1.5 g for an infusion period of more than 1 hour, for treatment of pneumonia a dosage of 3x 3 g is used in the approval study (https://clinicaltrials.gov/ct2/show/NCT02070757).

### Ertapenem

Ertapenem is a carbapenem with no activity against non-fermenters and enterococci. In contrast to the US approval, ertapenem is only approved in Europe for the treatment of intra-abdominal infections, community-acquired pneumonia, acute gynecological infections and cutaneous and soft tissue infections in diabetic foot syndrome but not for urinary tract infections and pyelonephritis, although good data are available [25]. Ertapenem, like all carbapenems, is active against ESBL-positive enterobacteria. Due to the long half-life, the dosage is 1x 1 g of ertapenem, whereas for the treatment of non-urogenital infections the off-label use of 1x 2 g of ertapenem is recommended because of the high protein binding [26]. Caution is advised in patients with impaired renal function, as they may experience class-typical CNS effects due to higher plasma levels.

### Tigecycline

Tigecycline, an advanced form of minocycline, is the first broad-spectrum glycylcycline which is also effective against MRSA, VRE and ESBL-producing Enterobacteriaceae. However *Pseudomonas* *aeruginosa* is not only unaffected but even selected. 

Due to the excellent membrane permeability and the associated high volume of distribution, tigecycline only achieves very low serum concentration. Thus the substance is only partly suitable for the treatment of bacteremic infections [27]. In clinical trials it was significantly inferior to imipenem in the treatment of patients with *Acinetobacter baumannii* bacteremia [28]. Tigecycline was also significantly inferior to imipenem in the treatment of patients with nosocomial, ventilator-associated pneumonia [29], [30]. The reason for this may have been an excessively low AUC/MIC ratio due to only moderate pulmonary penetration. In a Phase II trial, tigecycline was used in higher doses in patients with nosocomial pneumonia. Clinical cure success was higher in the group of patients receiving 200 mg as the initial dose and 100 mg every 12 hrs thereafter than in the group of patients treated with imipenem and the lower-dose tigecycline group (150 mg as the starting dose, thereafter 75 mg every 12 hrs) [31]. Therefore, in severe infections, tigecycline should be used at the higher dose described [31].

### Fosfomycin

Fosfomycin should only be given in combination treatment and, with regard to the PK/PD ratio, in high doses (up to 24 g fosfomycin/day, see Table 2 (Tab. 2) and Table 3 (Tab. 3)) in order to avoid the risk of developing resistance during treatment [32], [33]. Fosfomycin has numerous benefits such as lack of protein binding, high levels of activity and very good penetration into muscle, lungs, bones, cerebrospinal fluid [34] and biofilms as well as protection against ototoxicity and nephrotoxicity [35]. The high sodium load (14.5 m Na^+^ per g) and the increased potassium secretion are negative aspects. 

An early meta-analysis examined the efficacy of fosfomycin in ESBL-positive strains of *Escherichia coli* and *Klebsiella pneumoniae* respectively and concluded that fosfomycin may be used in urinary tract infections [36]. However, a Spanish study published in the same year reported that increasing numbers of fosfomycin prescriptions were connected to an increase in the rate of ESBL-positive *Escherichia coli* strains resistant to fosfomycin from 4.4% (2005) to 11.4% (2009) [37]. 

### Colistin

Regarding dosages, it should be noted that 30 mg colistin base corresponds to 1 million IU. Colistin is active against both ESBL-producing Enterobacteriaceae and carbapenemase-producing Enterobacteriaceae in vitro. Colistin is also active against carbapenem-resistant *Acinetobacter baumannii* strains and against multidrug-resistant *Pseudomonas aeruginosa* isolates.

Colistin is indicated for the treatment of the following infections: ventilator-associated pneumonia, bacteremia/sepsis, abdominal, urinary tract and bone infections as well as meningitis [38]. The initial i.v. loading dose should be 9–12 million IU [39], since otherwise sufficiently high levels of effectiveness can only be achieved after 2–3 days. Higher maintenance doses are usually well tolerated taking into account body weight, creatinine clearance and neurotoxicity [39], [40].

In addition to systemic administration, there is the option of inhaled administration for the treatment of pneumonia. Significantly higher concentrations are achieved in sputum [41] and lung tissue [42], [43] compared to intravenous administration. Inhalation therapy as an addition resulted in faster microbiological eradication and higher clinical healing rates. However, no reduction in lethality has been demonstrated in clinical studies to date [44], [45]. Application should be carried out using an ultrasonic nebulizer with a particle size of 3–5 µm [46]. 

Due to the very poor penetration of polymyxins into the CNS when given intravenously, colistin can be administered intraventricularly or intrathecally in patients with CNS infections. 

## Treatment of infections with extended spectrum beta-lactamase-producing Enterobacteriaceae

**Figure 1:**
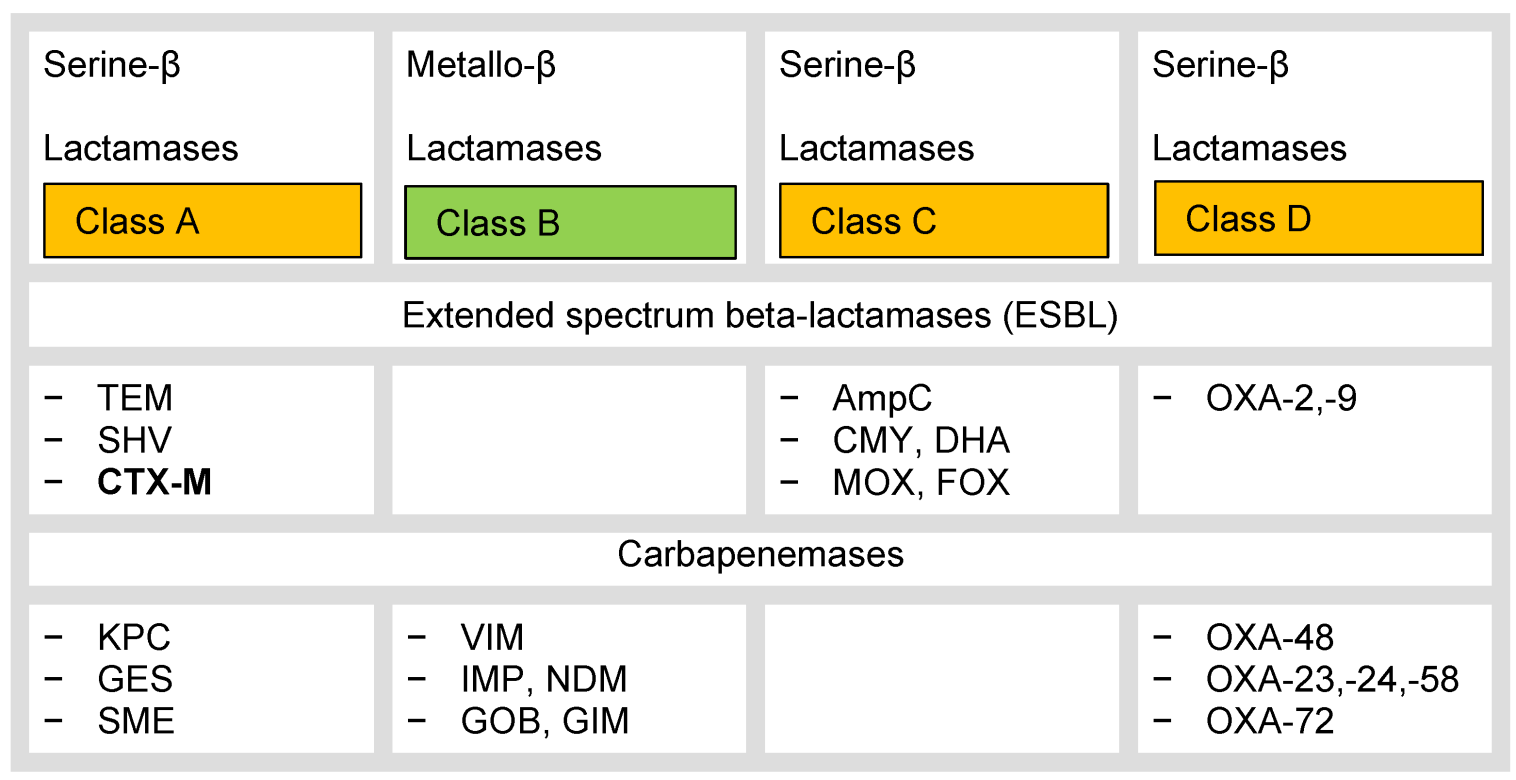
Classification of beta-lactamases, according to [48]

Extended-spectrum beta-lactamase producing enterobacteria have become increasingly important in recent years and present a major therapeutic problem in Europe today compared to methicillin-resistant *Staphylococcus aureus* strains [47]. The beta-lactamases can be divided phenotypically into four classes according to the Ambler classification (A–D, Figure 1 (Fig. 1)) [48] or functionally into three groups according to Bush-Jacoby. Class A and D enzymes hydrolyze penicillins and, to a lesser extent, oxyimino-cephalosporins; class C beta-lactamases hydrolyze cephalosporins more than penicillins [49]. Awareness of the individual beta-lactamases is necessary in view of the new treatment options in order to apply the new cephalosporin combinations precisely and to save carbapenems in the treatment of ESBL-positive enterobacteria; these are often resistant to fluoroquinolones.

Treatment options currently include beta-lactamase inhibitors (avibactam, clavulanic acid, tazobactam) in fixed combination with penicillin (amoxicillin/clavulanic acid, piperacillin/tazobactam) or cephalosporin (ceftazidime/avibactam, ceftolozane/tazobactam) as well as temocillin, carbapenems (ertapenem, imipenem/cilastatin, meropenem), colistin, fosfomycin, and tigecycline. 

## Treatment of infections with carbapenemase-producing Enterobacteriaceae

The treatment of infections with carbapenemase-producing Enterobacteriaceae, especially *Klebsiella pneumoniae* but also *Escherichia coli* and other representatives, so-called 4MRGN, is characterized by extremely limited treatment options and the absence of prospective randomized multicenter studies. Two prospective randomized studies are currently investigating colistin in monotherapy versus colistin in combination with a carbapenem (NCT01732250 and NCT01597973). The results of these studies were not available at the time of publication of these recommendations [50]. Therefore the current treatment recommendations are based essentially on case series, observational studies, non-randomized comparative studies and expert opinions and focus on infections with *Klebsiella pneumoniae*, usually with *Klebsiella* *pneumoniae* carbapenemases (KPC), OXA-48 or metallo-beta-lactamases (for example VIM). Whether the results are transferable to other Enterobacteriaceae with carbapenem resistance and other mechanisms of carbapenem resistance is currently unclear. 

The prevalence of carbapenem-resistant Klebsiella is also increasing slowly in Germany but is still very low. The Antibiotic Resistance Surveillance System (ARS) at the RKI reports a prevalence of 0.4% carbapenem-intermediate and -resistant strains for imipenem and meropenem in 2015 (https://ars.rki.de/Content/Database/ResistanceDevelopment.aspx). In the 2013 PEG Resistance Study, the proportion of strains that were no longer sensitive was 1.6% (imipenem) and 1.3% (meropenem) (https://www.p-e-g.org/resistenzdaten.html). Data from the National Reference Center for Gram-negative Pathogens show that OXA-48 is found in Germany, as well as KPC-2, VIM-1, NDM-1 and KPC-3 [51].

In principle, colistin, tigecycline, some aminoglycosides and fosfomycin are available as treatment options effective in vitro. Ceftazidime/avibactam is also active against KPC-producers in vitro. However, the status of ceftazidime/avibactam as a potential treatment option for infections with KPC producers can currently not be assessed due to limited clinical data.

The detection of a carbapenemase as a resistance mechanism does not always lead to a phenotypically resistant pathogen. Therefore knowledge of the minimum inhibitory concentration (MIC) of the pathogen is essential and the reason why microbiological laboratories should definitely report the MIC for imipenem and/or meropenem in carbapenemase producers. While the MIC for ertapenem is the best marker for the presence of carbapenemase, it plays a minor role in deciding options for combination therapy. Compared to meropenem, efficacy as a combination partner is postulated for MIC values up to 8 mg/l.

However, clinical data available to date from non-randomized small case studies indicate that combination treatment involving carbapenem should be preferred for infections with carbapenem-resistant Enterobacteriaceae [52], [53], [54]. However, there are many unanswered questions regarding the data. In a case series from Greece [52] many isolates were phenotypically non-carbapenem-resistant and, in most cases, no information was provided whether the patients had mono-infection by the carbapenem-resistant strain or polymicrobial infection involving carbapenem-sensitive isolates. This would create a bias in favor of the combination as the carbapenem-sensitive pathogens were treated with an effective treatment regimen [55]. In most studies, no adjustment was made as to whether the calculated initial treatment was adequate or inadequate. Another critical point is that the dose recommendations for colistin have recently been increased significantly. In the “Combination Therapy Studies”, the dosages administered were not sufficiently high according to today’s standards. 

A therapeutic approach with two carbapenems – ertapenem plus doripenem or meropenem – is theoretically attractive [56]. The principle is based on the fact that the carbapenemases have a higher affinity for ertapenem than for doripenem and meropenem. When ertapenem is given (1 hour) before doripenem or meropenem, ertapenem is inactivated but remains bound to the carbapenemase, so that the other carbapenem (doripenem or meropenem) may act [56]. To date, 38 cases of patients receiving this treatment regimen have been published. In 22 patients treatment was successful [56], [57], [58], [59].

A multi-center prospective observational study with 41 intensive care patients is available on the importance of fosfomycin as a combination partner. In all cases, a bloodstream infection or ventilator-associated pneumonia with a carbapenem-resistant *Klebsiella pneumoniae* strain had been diagnosed. Treatment led to clinical success in approximately 54% of patients given a median dose of 24 g/day. The combination partners were predominantly tigecycline and colistin but also carbapenems and aminoglycosides [60].

To date there is no data available on a suitable combination of antibiotics to be used as a “carbapenem-sparing” regimen or in situations where carbapenem cannot be used because of the level of MICs. It is also unclear whether a combination of three in vitro antibiotics is superior to a combination of two in vitro antibiotics, although the data from some case series could be cautiously interpreted in this direction. 

### Recommended treatment

On the basis of the available data, despite the low level of evidence, a recommendation for treatment was issued by the expert group, which should serve as the basis for the treatment of patients with severe infections with carbapenem-resistant Enterobacteriaceae until the results of randomized clinical studies are available.

Combination treatment is recommended for severe infections such as bloodstream infections or pneumonia. If carbapenem MIC is at or below 8 mg/l, carbapenem-based combination treatment is preferable. If the carbapenem MIC is above 8 mg/l, a combination of colistin and tigecycline and, if appropriate, fosfomycin or an aminoglycoside should be given.

Prolonged or continuous administration of carbapenems for the treatment of infections by carbapenem-resistant Enterobacteriaceae has not been studied to date. Therefore, no recommendation is made here for or against these forms of application. However, continuous administration of a carbapenem should never take place without therapeutic drug monitoring, since there is a risk of continuous sub-therapeutic levels (see also chapter 3 [61] and chapter 11 [62]). 

Colistin achieves only low concentrations in the lung tissue when administered systemically, so that inhalation therapy as an addition can be considered in pneumonia [63].

Table 3 (Tab. 3) provides suggestions for the treatment of pneumonia and sepsis by carbapenem-resistant Enterobacteriaceae.

## Treatment of infections with carbapenem-resistant Acinetobacter baumannii strains

The treatment of infections with carbapenem-resistant *Acinetobacter baumannii* strains, so-called 4MRGN, presents a great challenge. In these cases, only a few effective antibiotics are available and for which there are no large-scale prospective studies on clinical efficacy. So the treatment recommendations are based on case series, non-randomized comparative studies and expert opinions.

Apart from colistin and tigecycline, sulbactam and cotrimoxazole are important in *Acinetobacter baumannii* infections.

Colistin should be used in combination with a second active substance, for example tigecycline, sulbactam, an aminoglycoside or even a carbapenem because smaller observational studies have provided evidence that combination treatment is superior to monotherapy with colistin [64].

### Sulbactam

Sulbactam has a high affinity for the penicillin-binding proteins 1a and 2 and therefore as the only synthetic beta-lactamase inhibitor has relevant antibacterial activity against *Acinetobacter baumannii*. The substance is characterized as a time-dependent bactericide, which is best described by the %T >MIC [65], [66]. In animal experiments treatment with sulbactam showed results comparable to imipenem but higher healing and survival rates compared to colistin [67]. The few clinical data available indicate that treatment with sulbactam is as effective as treatment with a carbapenem or colistin [68], [69], [70]. Another study even found significantly higher clinical healing rates for sulbactam compared to colistin [71].

### Cotrimoxazole (trimethoprim/sulfamethoxazole)

Cotrimoxazole shows high in vitro efficacy, even in colistin-resistant strains [72], [73]. 92.1% of the *Acinetobacter baumannii* complex isolates tested by ARS in Germany in 2015 were cotrimoxazole-sensitive [74]. Comparable data were found in the PEG resistance studies in 2010 and 2013 with sensitivity rates of 74.7% and 71.6% respectively for *Acinetobacter baumannii* sensu stricto, [75]. However, there is no clinical study on efficacy. There are only case reports where cotrimoxazole was usually given in combination with a second substance. All published cases of treatment with cotrimoxazole have been described as a therapeutic success [76]. A general treatment recommendation cannot be given for lack of data. However, cotrimoxazole remains a possible treatment option, especially for infections caused by colistin-resistant strains and particularly in urinary tract infections.

### Combination treatment

Several combination treatments were investigated in multiple studies due to the sub-optimal pharmacokinetics and rapid development of resistance both to colistin and tigecycline and the limited predictability of the results of in vitro testing of sulbactam on clinical efficacy. In a retrospective study of patients with *Acinetobacter baumannii* bacteremia, the combination of colistin either with a carbapenem, sulbactam or in a few patients with another combination partner, was significantly superior to colistin monotherapy as regards mortality [64]. Carbapenems appear to be clinically synergistic in combination with colistin even with in vitro resistance. 

Rifampicin shows high activity in vitro against multidrug-resistant *Acinetobacter baumannii* strains. In animal experiments, a superiority of the combination of rifampicin with colistin was demonstrated in comparison to colistin alone [64]. However, this effect could not be confirmed in two prospective clinical studies [77], [78]. Therefore, combination treatment with rifampicin is currently not recommended due to the high potential for interaction and hepatotoxicity [79].

Table 4 (Tab. 4) and Table 5 (Tab. 5) summarize the proposals for the treatment of infections with carbapenem-resistant *Acinetobacter baumannii*.

## Note

This is the sixteenth chapter of the guideline “Calculated initial parenteral treatment of bacterial infections in adults – update 2018” in the 2^nd^ updated version. The German guideline by the Paul-Ehrlich-Gesellschaft für Chemotherapie e.V. (PEG) has been translated to address an international audience.

## Competing interests

The authors declare that they have no competing interests.

## Figures and Tables

**Table 1:**
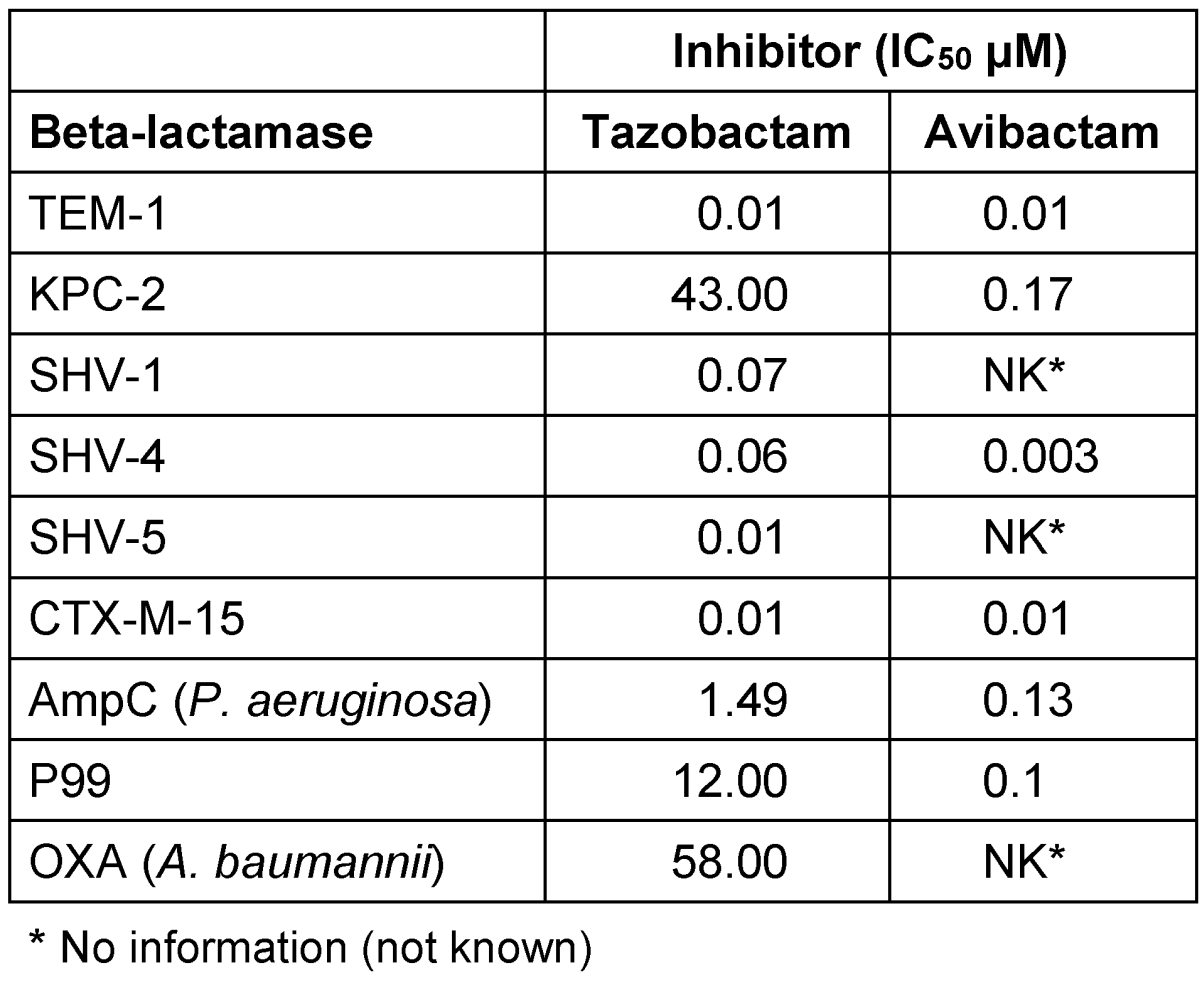
Activity of beta-lactamase inhibitors [15]

**Table 2:**
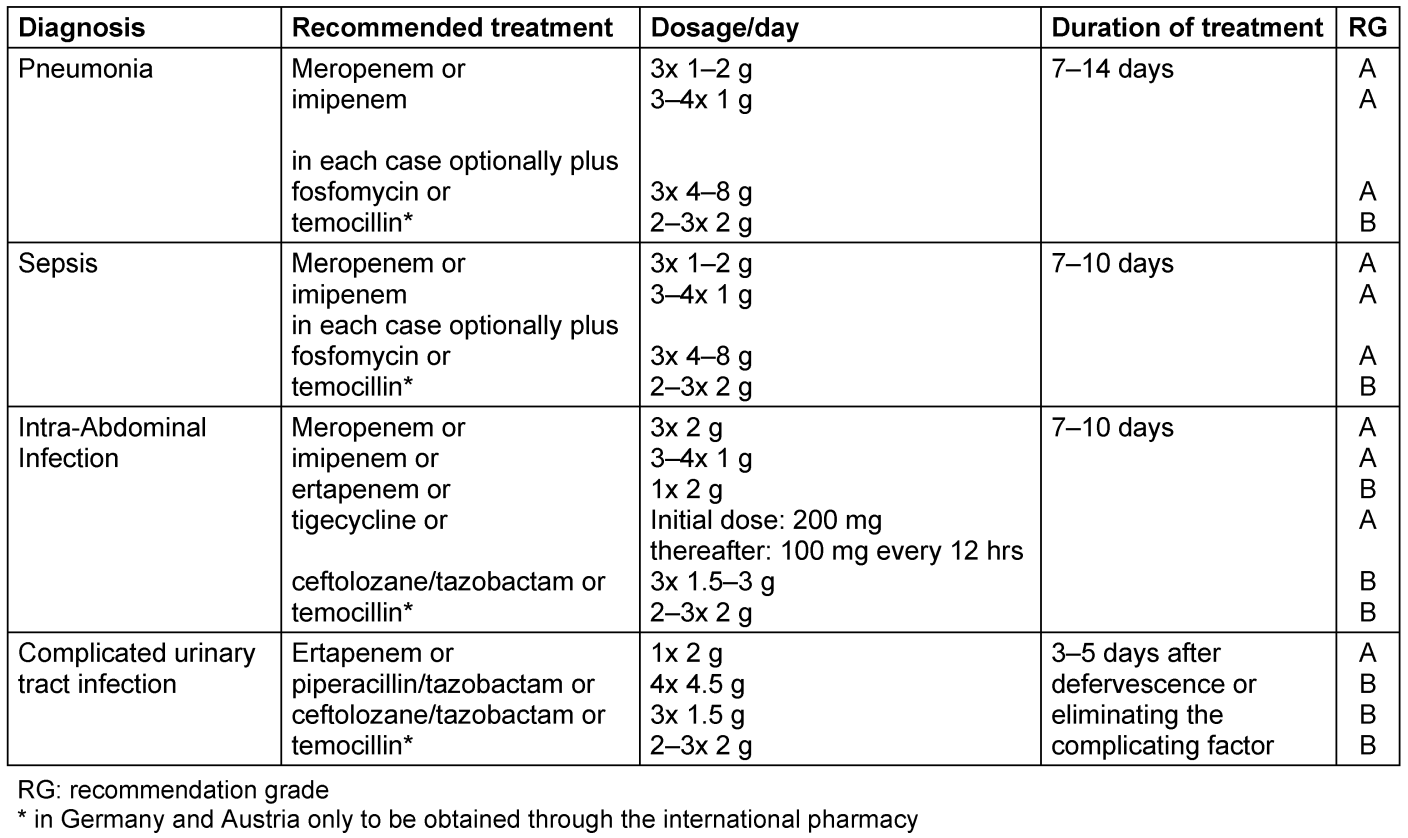
Treatment recommendations for infections with ESBL-producing Enterobacteriaceae

**Table 3:**
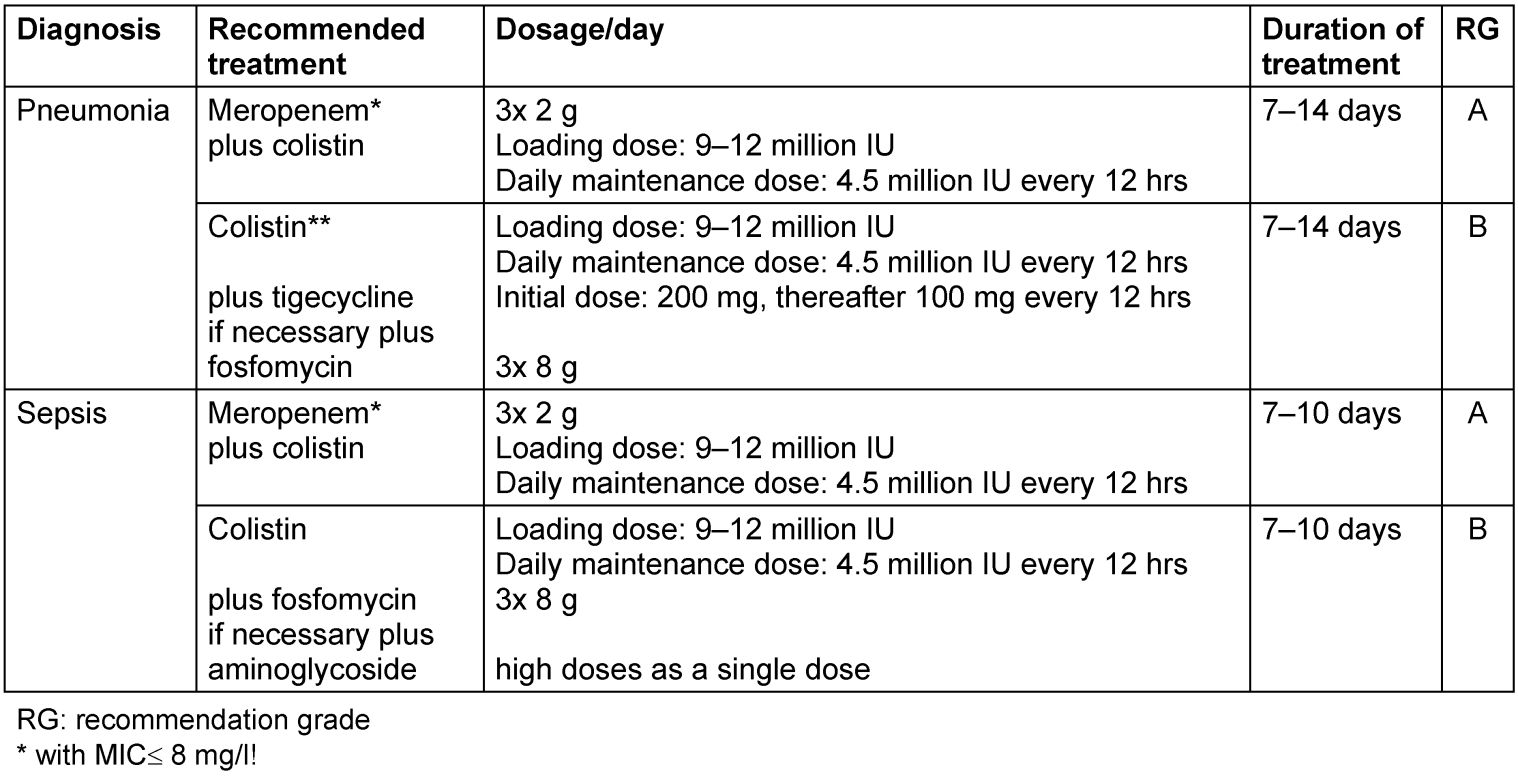
Treatment recommendations for carbapenem-resistant Enterobacteriaceae

**Table 4:**
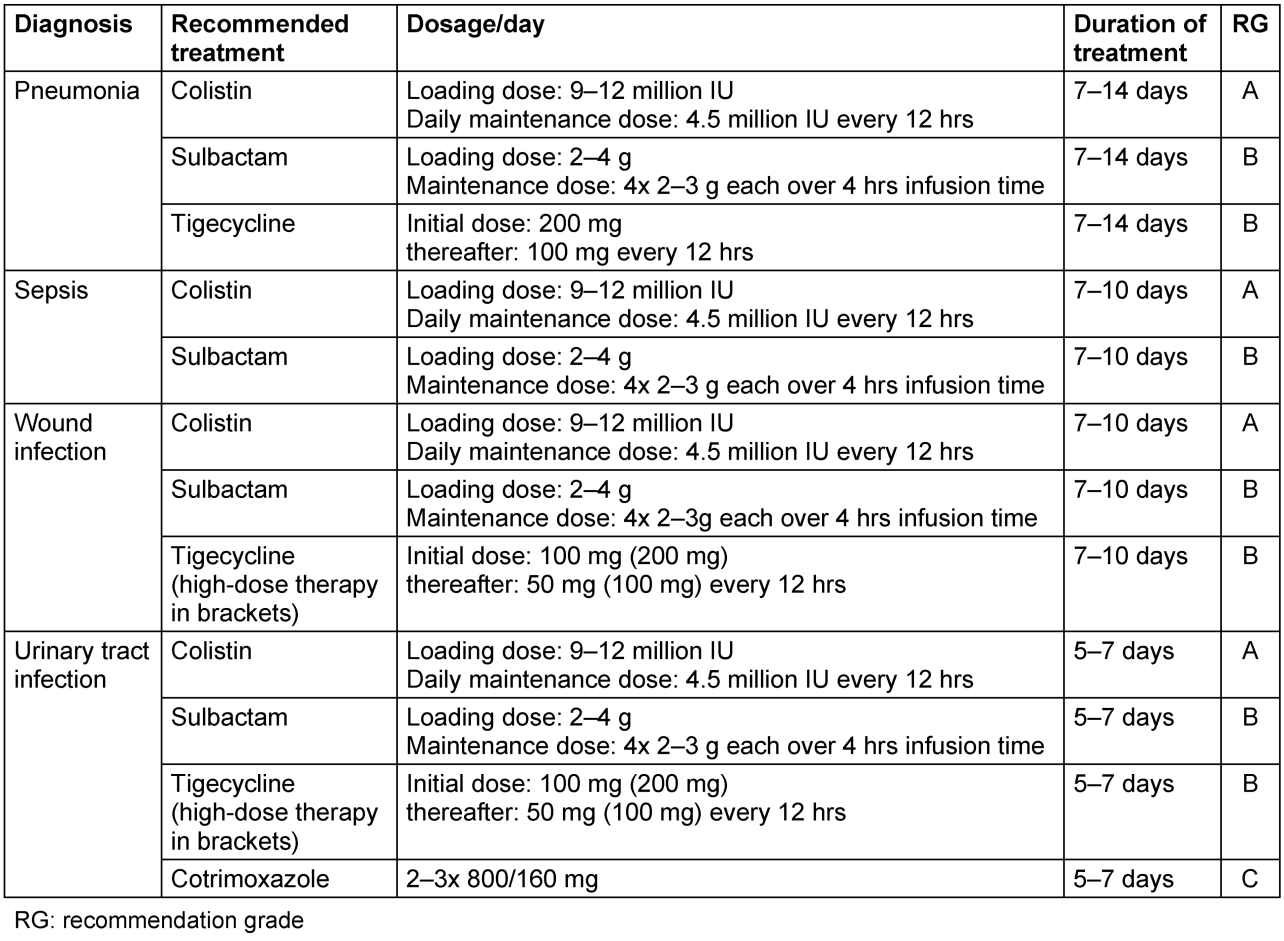
Treatment of infections with carbapenem-resistant Acinetobacter baumannii

**Table 5:**
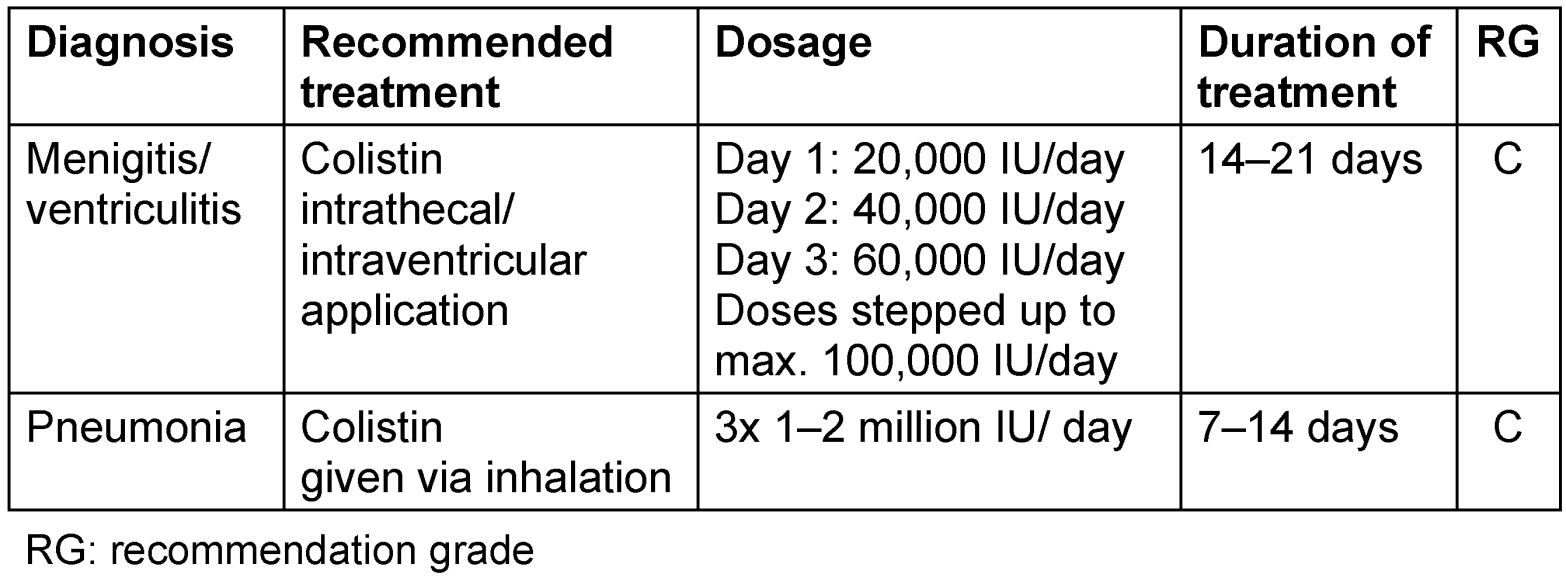
Special application forms of colistin
